# Vitamin D signaling increases nitric oxide and antioxidant defenses of bovine monocytes

**DOI:** 10.3168/jdsc.2020-0005

**Published:** 2021-01-22

**Authors:** Mercedes F. Kweh, Kathryn E. Merriman, Teri L. Wells, Corwin D. Nelson

**Affiliations:** 1 Animal Molecular and Cellular Biology Graduate Program, University of Florida, Gainesville 32611; 2 Department of Animal Sciences, University of Florida, Gainesville 32611

## Abstract

Vitamin D contributes to multiple aspects of bovine immunity and is reported to decrease the effects of mastitis and metritis in dairy cows. We hypothesized that vitamin D signaling in bovine monocytes increases antioxidant responses as part of its immunomodulatory actions. Our objectives were to assess the effects of vitamin D on oxidant and antioxidant responses of bovine monocytes. Monocytes from peripheral blood of nonpregnant, lactating Holstein cows between 90 and 300 d in milk were used for in vitro cell culture experiments. To test the effects of vitamin D on reactive oxygen metabolites (dROM) and antioxidant potential (AOP), monocytes from 14 cows were cultured in replicates for 16 h with 25-hydroxyvitamin D_3_ [25(OH)D_3_, 0 or 75 ng/mL] in a factorial arrangement with lipopolysaccharide (LPS, 100 ng/mL) or interferon-γ (IFN-γ, 10 ng/mL) or with no stimulation. Data were analyzed by ANOVA for main effects of 25(OH)D_3_, stimulant, and interactions between 25(OH)D_3_ and stimulant. Significant interactions between 25(OH)D_3_ and stimulant were observed for dROM and AOP of culture supernatants. In unstimulated cultures, 25(OH)D_3_ tended to increase dROM, but the opposite was observed in stimulated cultures. In contrast, LPS and IFN-γ treatments alone decreased AOP of culture supernatants, but 25(OH)D_3_ counteracted the decrease in AOP caused by IFN-γ. Abundances of transcripts of genes encoding antioxidant-related proteins were measured by quantitative PCR using RNA from monocytes from 4 cows treated with 25(OH)D_3_ (0 or 75 ng/mL) in a factorial arrangement with increasing concentrations of LPS (0 to 1,000 ng/mL) or IFN-γ (0 to 10 ng/mL). Treatment with 25(OH)D_3_ increased transcripts of genes encoding metallothionein 1A and metallothionein 2A in the presence of IFN-γ but not LPS. Furthermore, 25(OH)D_3_ increased transcripts of genes encoding thioredoxin and thioredoxin reductase, but the effect of 25(OH)D_3_ did not depend on IFN-γ or LPS stimulation. In conclusion, 25(OH)D_3_ increased antioxidant capacity of IFN-γ–stimulated bovine monocytes, potentially by increasing metallothionein and thioredoxin activities in monocytes.

## Highlights

• Vitamin D and interferon-gamma (IFN-γ) increased monocyte nitric oxide production
• IFN-γ decreased antioxidant potential of monocyte cultures
• Vitamin D signaling increased antioxidant potential of IFN-γ-stimulated monocytes
• Vitamin D increased abundance of metallothionein and thioredoxin transcripts

## Graphical Abstract

**Figure:**
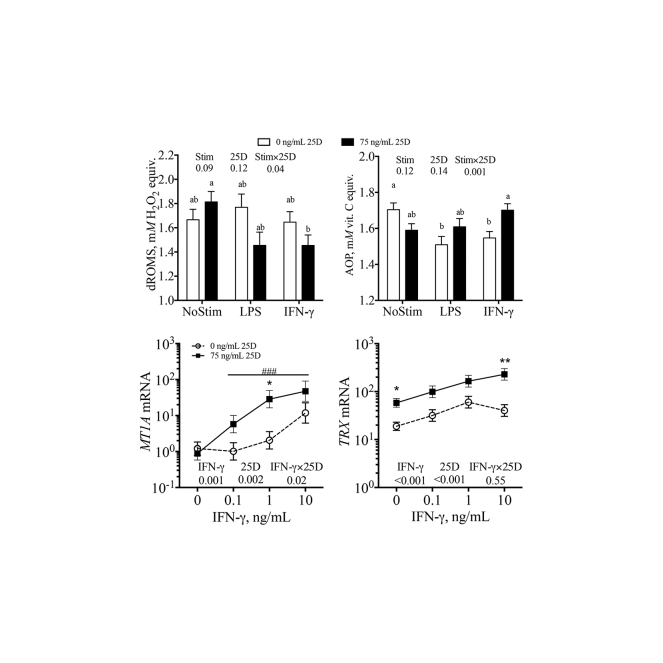
**Summary:** The oxidative burst of phagocytes is a key part of antimicrobial defenses. Vitamin D contributes to several aspects of bovine immunity, including antimicrobial activity. Our objectives were to assess the effects of vitamin D on the oxidant and antioxidant responses of freshly isolated monocytes of dairy cows. Vitamin D increased antioxidant potential of interferon-γ-stimulated monocyte cultures. Vitamin D also increased mRNA transcripts for metallothionein (*MT1A*) and thioredoxin (*TRX*) genes. Our data indicate a potential role for vitamin D in maintaining redox balance during infection.

Recent reports have documented positive effects of 25-hydroxyvitamin D_3_ [**25(OH)D_3_**] for protection against uterine and mammary infections in dairy cows (Lippolis et al., 2011; Martinez et al., 2018; Poindexter et al., 2020). Concentrations of 25(OH)D_3_ in serum of dairy cows during the postpartum period, a time when cows are at greatest risk of disease, are lower compared with those during prepartum or mid lactation (Nelson et al., 2016; Holcombe et al., 2018). Moreover, Wisnieski et al. (2020) reported that cows with serum 25(OH)D_3_ >71 ng/mL in the postpartum period were at lowest risk for uterine diseases.

The direct actions of vitamin D in the immune system of cows provide a likely explanation for the positive effects of 25(OH)D_3_ in reduction of dairy cow diseases. Toll-like receptor agonists such as LPS and IFN-γ stimulate an intracrine vitamin D pathway in innate immune cells that contributes to activation of multiple immune functions. For example, intramammary LPS challenge increased transcripts for 1α-hydroxylase (**CYP27B1**), which catalyzes conversion of 25(OH)D_3_ to 1,25-dihydroxyvitamin D_3_ [**1,25(OH)_2_D_3_**], and the vitamin D receptor (**VDR**) in macrophages and neutrophils in the mammary gland (Merriman et al., 2018). The effects of vitamin D signaling in immunity are quite diverse and include induction of antimicrobial responses and chemokines, and suppression of proinflammatory T cells and cytokines (Hewison, 2012). In particular, 1,25(OH)_2_D_3_ elicits a robust nitric oxide (NO) response in bovine monocytes and macrophages, which enhances killing of *Mycobacteria bovis* by macrophages (García-Barragán et al., 2018). Intramammary and dietary vitamin D treatments also increased abundance of transcripts for inducible nitric oxide synthase (**NOS2**) in immune cells of the mammary glands of cows (Merriman et al., 2017; Poindexter et al., 2020). However, NO production by immune cells may increase oxidation of host cell membranes and proteins and, if not balanced by protective antioxidant mechanisms, lead to tissue damage (Shi et al., 2018). In addition to the effects of vitamin D on nitric oxide, it also is known to have positive effects on antioxidant status in human and rodent cells. We hypothesized that vitamin D signaling in bovine monocytes would increase antioxidant responses to counteract pro-oxidant responses to LPS or IFN-γ. Therefore, our objectives were to assess the effects of vitamin D signaling on oxidant and antioxidant responses of bovine monocytes.

Monocytes used in the experiments were collected from lactating, nonpregnant Holstein cows at the University of Florida Dairy Unit according to approval of the University of Florida Institutional Animal Care and Use Committee. Average ± SD parity, days in milk, and milk yield of cows were 1.7 ± 0.9 parities, 207 ± 99 d, and 29.5 ± 9.4 kg/d, respectively. Cows were free of any clinical diseases before and at the time of blood collection. Cows were fed a standard lactating cow TMR that provided cows with approximately 40,000 IU of vitamin D_3_/d.

Monocytes were isolated as previously described (Merriman et al., 2015). Briefly, 50 mL of blood was sampled from the jugular vein using 60-mL syringes containing 5 mL of acid citric dextrose solution. Blood was centrifuged at 1,500 × *g* for 20 min, the buffy coat layer was collected, and erythrocytes were removed by hypotonic lysis. Mononuclear cells were then layered over 1.083 g/mL Percoll and centrifuged for 30 min at 450 × *g* to remove any remaining neutrophils. Monocytes were isolated by adherence on a T-75 tissue-culture-treated flask for 1 h in RPMI 1640 medium (Hyclone Laboratories, Logan UT) containing 10% fetal bovine serum (characterized; Hyclone Laboratories). After removal of non-adherent cells by washing with warm Dulbecco's PBS, monocytes were collected from the flask with ice-cold PBS. Monocytes were counted, resuspended in RPMI 1640 medium containing penicillin-streptomycin (100 units each/mL) and 10% fetal bovine serum to a concentration of 1 × 10^6^ cells/mL. Finally, 200 μL of cell suspensions were added to 96-well tissue culture-treated plates before applying treatments. All cell culture reagents, unless otherwise noted, were purchased from Fisher Scientific (Waltham, MA). The 25(OH)D_3_ and 1,25-dihydroxyvitamin D_3_ were purchased from Cayman Chemical (Ann Arbor, MI) and dissolved in reagent-grade ethanol. Lipopolysaccharide derived from *Serratia marcescens* was purchased from Sigma Aldrich (St. Louis, MO). Recombinant bovine IFN-γ was purchased from R&D Systems (Minneapolis, MN).

Each experiment was a randomized, complete block design with cow (the source of monocytes) as the blocking factor. For experiments used to test effects of 25(OH)D_3_ on nitrite (experiment 1), reactive oxygen metabolites and antioxidant potential (experiment 2), 2 levels of 25(OH)D_3_ (0 or 75 ng/mL) and 3 types of stimulation (no stimulation, 100 ng/mL LPS, or 10 ng/mL IFN-γ) were applied in a factorial arrangement. Experiment 1 was replicated with monocytes from 7 cows, whereas experiment 2 was replicated with monocytes from 14 cows. Experiment 3 tested the effects of 1,25(OH)_2_D_3_ on monocyte gene expression. Monocytes from 4 cows were stimulated with 0 or 100 ng/mL LPS and 0 or 4 ng/mL of 1,25(OH)_2_D_3_ in a factorial arrangement. Experiment 4 tested the effect of 25(OH)D_3_ in combination with LPS or IFN-γ. Monocytes from 4 cows were treated with 25(OH)D_3_ at 0 or 75 ng/mL in a factorial arrangement with 4 levels of LPS (0, 10, 100, and 1,000 ng/mL) or IFN-γ (0, 0.1, 1, and 10 ng/mL). For each experiment, monocyte cultures were incubated for 16 h in a humidified CO_2_ incubator at 37°C with 5% CO_2_.

Concentrations of nitrite in cell culture supernatants were measured using the Griess assay (0.5% sulfanilamide, 2.5% phosphoric acid, and 0.05% *N*-naphthyl-ethylenediamine dihydrochloride; Sigma) as previously described (Nelson et al., 2010). Nitrite is generated from NO in aerobic aqueous solutions, whereas, peroxynitrite is generated from reaction of NO with superoxides in diffusion-limited environments (Ignarro et al., 1993). Concentrations of reactive oxygen metabolites (**dROM**) and antioxidant potential (**AOP**) of cell culture supernatants were measured using the FRAS-5 system with Redox Fast kits (H&D S.R.L. Str. Langhirano, Parma, Italy) according to the manufacturer's instructions. The assays were performed immediately after collection of culture supernatants. The dROM assay measures hydroperoxides by photometric measurement of oxidized diethyl-*para*-phenylenediamine (**DEPPD**) absorption at 505 nm. The dROM assay is not known to distinguish between reactive nitrogen species (**RNS**) and reactive oxygen species (**ROS**) derivatives (Alberti et al., 2000). Briefly, 10 μL of culture supernatant was added to the dROM kit reagent containing DEPPD, mixed gently for 10 s, and measured with the FRAS-5 photometer. Likewise, the AOP assay measures capacity to reduce ferric iron in the sample by photometric absorption at 505 nm. Ten microliters of supernatant was added to the AOP reagent, mixed gently for 10 s, and measured with the FRAS-5 photometer. The dROM and AOP are reported in equivalents of H_2_O_2_ and ascorbic acid, respectively.

The oxidative burst capacity of monocytes was measured using dihydrorhodamine 123 (**DHR**, Sigma). Reactive nitrogen species (i.e., peroxynitrite) and ROS, but not NO, will oxidize DHR to rhodamine (Crow, 1997). After culture with treatments for 16 h, monocytes were removed from the cell culture plate, resuspended in 100 µL of fresh culture medium, and incubated with 10 µL of 50 µ*M* DHR for 10 min at 37°C. Eight million colony-forming units of heat-killed *Escherichia coli* O8:H19, prepared as previously described (Martinez et al., 2018), was then added to monocytes, resulting in 40 *E. coli* per monocyte to stimulate oxidative burst in monocytes. Samples incubated for 30 min at 37°C with continuous mixing were then analyzed using an Accuri C6 flow cytometer (Becton Dickinson). Median fluorescence intensities (**MFI**) of DHR in 2,500 cells were determined with FCS Express 6.0 (De Novo Software, Pasadena, CA).

Total RNA of monocytes was isolated using the Quick-RNA MiniPrep RNA isolation kit (Zymo Research, Irvine, CA) as per the manufacturer's instructions and eluted with 80 μL of nuclease-free water. The RNA (10 μL at 9 ± 5 ng/µL, 260/280 ratio of 1.8 ± 0.3) was reverse transcribed using the High Capacity cDNA Reverse Transcription Kit (Life Technologies, Carlsbad, CA) in a 20-µL reaction as per the manufacturer's instructions with random primers and 1 μL of RNase inhibitor (RiboLock, Thermo Fisher Scientific, Waltham, MA). The reverse transcription reaction was incubated in a thermal cycler (Eppendorf, Hamburg, Germany) for 10 min at 25°C, 60 min at 37°C, and 5 min at 85°C. Quantitative PCR was performed as previously described (Kweh et al., 2019) using a CFX96 Touch Real-Time PCR Detection System (BioRad, Hercules, CA) with 20-μL reactions containing 9 μL of cDNA, 0.5 μL each of forward and reverse primers, and 10 μL of SYBR Select qPCR Master Mix (ThermoFisher). Primer sequences for β-actin (*ACTB*), *GAPDH*, ribosomal protein S9 (*RPS9*), 1α-hydroxylase (*CYP27B1*), 24-hydroxylase (*CYP24A1*), β-defensin 7 (*DEFB7*), inducible nitric oxide synthase (*NOS2*), glutathione peroxidase 1 (*GPX1*), metallothionein 1A (*MT1A*), metallothionein 2A (*MT2A*), nuclear factor erythroid 2-related factor 2 (*NFE2L2*), thioredoxin (*TRX*), and thioredoxin reductase 1 (*TXNRD1*) genes are provided in Table 1. The threshold cycle (**Ct**) for each gene was normalized to the geometric mean of *ACTB, GAPDH*, and *RPS9* Ct values using the equation ΔCt = Ct_(target gene)_ – Ct_(reference genes)_. The ΔCt values for each gene were used for statistical analysis.

Data were analyzed by ANOVA using the GLIMMIX procedure in SAS (version 9.4, SAS Institute Inc., Cary, NC). Residuals were observed for normal distribution. The general mathematical model used for the analysis was Y_ijk_ = μ + B_i_ + L_j_ + D_k_ + L_j_D_k_ + e_ijk_, where Y_ijk_ = dependent variable, μ = overall mean, B_i_ = fixed effect of cow that was source of cells, L_j_ = fixed effect of stimulant or dose of stimulant, D_k_ = fixed effect of vitamin D, and e_ijk_ = residual error. For experiments 1 and 2, main effects of 25(OH)D_3_ (0 vs. 75 ng/mL), stimulant (no stimulant, LPS or IFN-γ), and interaction between stimulant and 25(OH)D_3_ were analyzed. The model for experiment 3 included effects of LPS (0 vs. 100 ng/mL), 1,25(OH)_2_D_3_ (0 vs. 4 ng/mL), interaction between LPS and 1,25(OH)_2_D_3_, and cow. Experiment 4 was performed as one experiment, but the effects of 25(OH)D_3_ were analyzed separately for LPS and IFN-γ treatments to account for the multiple doses of LPS and IFN-γ that were used. The model used for experiment 4 included effects of 25(OH)D_3_ (0 vs. 75 ng/mL), dose of stimulant (0 to 10 ng/mL IFN-γ or 0 to 1,000 ng/mL LPS), and interaction between 25(OH)D_3_ and dose of stimulant. Furthermore, the contrast statement was used to test the effect of 25(OH)D_3_ in the presence of stimulant (IFN-γ at 0.1, 1 and 10 ng/mL; LPS at 10, 100 and 1,000 ng/mL). Least squares means were computed for the interactions of 25(OH)D_3_ with LPS and IFN-γ, and the Tukey adjustment was applied to account for multiple comparisons of means. For gene expression data, least squares means of ΔCt values were transformed using the equation (2^−ΔCt^) and expressed as relative number of transcripts. Statistical significance was declared at *P* < 0.05 and tendencies were declared at *P* < 0.10 and *P* > 0.05.

Treatment of monocytes with IFN-γ, LPS, and 25(OH)D_3_ increased (*P* < 0.01) nitrite concentrations in culture supernatants (Figure 1A). However, none of the factors (LPS, IFN- γ, or 25(OH)D_3_) alone increased nitrite compared with cultures not stimulated and not treated with 25(OH)D_3_. Rather, nitrite was greater (*P* < 0.05) in cultures treated with LPS and 25(OH)D_3_ or IFN-γ and 25(OH)D_3_ compared with cultures that did not receive stimulant or 25(OH)D_3_ (Figure 1A). The IFN-γ treatment increased oxidative burst capacity, as measured by DHR in response to heat-killed *E. coli* challenge, but LPS and 25(OH)D_3_ treatments did not affect oxidative burst capacity of monocytes (Figure 1B). Interactions were observed between 25(OH)D_3_ and stimulation for dROM (*P* = 0.04) and AOP (*P* = 0.001). In the absence of LPS or IFN-γ, 25(OH)D_3_ tended to increase dROM in culture supernatants but had the opposite effect in the presence of LPS or IFN-γ stimulation such that dROM was lower (*P* < 0.05) in cultures treated with 25(OH)D_3_ and IFN-γ compared with 25(OH)D_3_ alone (Figure 1C). In contrast, 25(OH)D_3_ somewhat decreased AOP in the absence of IFN-γ or LPS but counteracted the decrease in AOP caused by stimulation, such that AOP of cultures treated with IFN-γ and 25(OH)D_3_ was greater (*P* = 0.04) than AOP of cultures treated with IFN-γ alone (Figure 1D).

**Figure 1:**
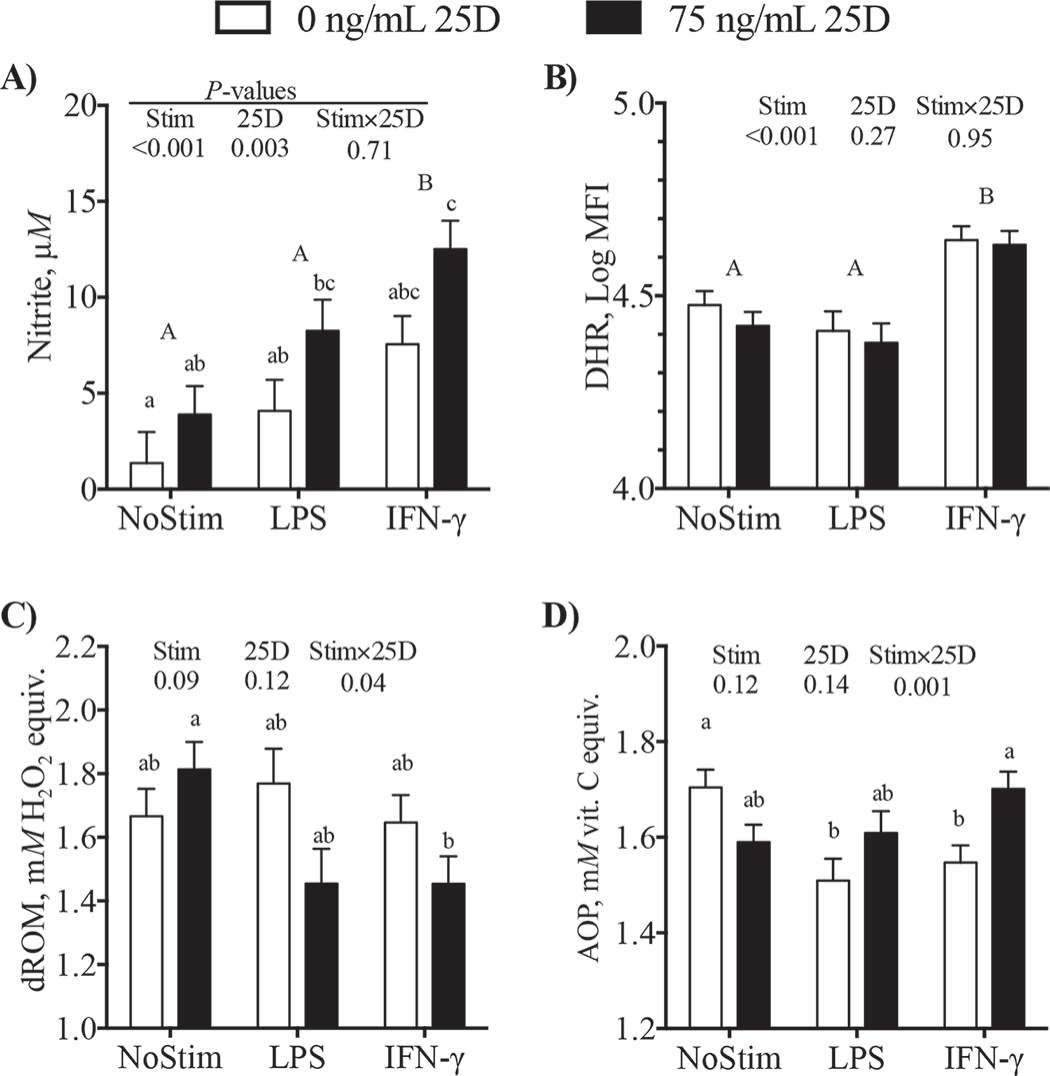
Monocytes from Holstein cows were treated with 0 or 75 ng/mL 25-hydroxyvitamin D_3_ (25D) in a factorial arrangement with no stimulant, 100 ng/mL LPS, or 10 ng/mL IFN-γ for 16 h. (A) Concentrations of nitrite in culture supernatant were measured using the Griess assay, n = 7. (B) Median fluorescence intensity (MFI) of monocyte oxidative burst capacity as measured by oxidation of dihydrorhodamine (DHR) using flow cytometry. After a 16-h culture period with treatments, DHR was added and oxidative burst was stimulated by addition of heat-killed *Escherichia coli*; n = 11. (C, D) Supernatants were collected and assessed for reactive oxygen metabolites (dROM) and antioxidant potential (AOP), n = 14. Values for dROM and AOP are reported as H_2_O_2_ and vitamin C equivalents, respectively. For all plots, data represent LSM ± SEM. The *P-*values for main effects of stimulant (IFN-γ, LPS or no stimulant), 25D (0 or 75 ng/mL), and their interaction (Stim × 25D) are indicated on each plot. The Tukey adjustment was applied to account for multiple means comparisons. Uppercase letters (A, B) indicate that LSM for main effect of stimulant are different (*P* < 0.05), and lowercase letters (a–c) indicate that LSM of individual treatments are different (*P* < 0.05).

We also evaluated the effects of vitamin D signaling on expression of antioxidant genes that may explain the changes in antioxidant potential of monocytes. Metallothionein and thioredoxin genes were increased by 1,25(OH)_2_D_3_ treatment according to RNA sequencing of monocytes treated with 1,25(OH)_2_D_3_ and LPS (C. D. Nelson, University of Florida, and J. D. Lippolis, USDA-ARS National Animal Disease Center, Ames, IA; unpublished data). Therefore, we hypothesized that vitamin D may increase expression of antioxidant genes in bovine monocytes. Because 1,25(OH)_2_D_3_ has more potent activity than 25(OH)D_3_ and it does not depend on CYP27B1 activity, the effects of vitamin D on expression of several genes encoding for antioxidant proteins were assessed in monocyte cultures treated with LPS and 1,25(OH)_2_D_3_ (Table 1). We found that 1,25(OH)_2_D_3_ increased, or tended to increase, transcripts of *GPX1, MT1A, MT2A, TRX*, and *TXNRD1* genes but not transcripts of *NFE2L2*. As positive controls of 1,25(OH)_2_D_3_ activity, 1,25(OH)_2_D_3_ also increased *CYP24A1, DEFB7*, and *NOS2* expression, as previously reported (Merriman et al., 2015).

**Table 1:** Effects of 1,25-dihydroxyvitamin D_3_ on expression of antioxidant genes

Gene	Primer sequence^1^	Fold change^2^	*P*-value^3^
*ACTB*	GGCATCCTGACCCTCAAGTA	—	—
	CACACGGAGCTCGTTGTAGA		
*GAPDH*	CCTGCCCGTTCGACAGATAG	—	—
	ATGGCGACGATGTCCACTTT		
*RPS9*	GTGAGGTCTGGAGGGTCAAA	—	—
	GGGCATTACCTTCGAACAGA		
*CYP24A1*	GAAGACTGGCAGAGGGTCAG	13.9	<0.01
	CAGCCAAGACCTCGTTGATT		
*CYP27B1*	TGGGACCAGATGTRRGCATTCGC	0.7	0.04
	TTCTCAGACTGGTTCCTCATGGCT		
*DEFB7*	TCTTCCTGGTCCTGTCTGCT	9.2	<0.01
	GGTGCCAATCTGTCTCCTGT		
*GPX1*	GCAACCAGTTTGGGCATCAG	1.8	0.02
	TAGGGTCGGTCATGAGAGCA		
*MT1A*	TCCCATCCGACCAGTGGATCT	1.8	0.06
	TTCTTGCAGGAGGGACATCTG		
*MT2A*	GCCATCCTTTGCTCAGCAGT	1.6	0.04
	GAGGCGCACTTGCAATCTTT		
*NOS2*	GATCCAGTGGTCGAACCTGC	9.9	<0.01
	CAGTGATGGCCGACCTGATG		
*NFE2L2*	GCATGATGGACTTGGAGCTG	0.9	0.44
	GCTCATGCTCCTTCTGTCGT		
*TRX*	ATTCCAACGTGGTGTTCCTTG	1.7	<0.01
	AGGTTGGCATGCATTTGACTT		
*TXNRD1*	AGGTCAAGCCCTACGAGACT	1.8	<0.01
	GCCCCAGTTGAGAGAACCAA		

1 Primer specificity and efficiency determined as previously described (Nelson et al., 2010). All primer pair efficiencies were >95%. Forward primer, top row; reverse primer, bottom row.

2 Monocytes were treated with 0 or 100 ng/mL LPS and 0 or 4 ng/mL 1,25-dihydroxyvitamin D_3_ [1,25(OH)_2_D_3_] in a factorial arrangement (n = 4 cows). Fold change represents main effect of 1,25(OH)_2_D_3_ compared with no 1,25(OH)_2_D_3_.

3 Significance of the main effect of 1,25-dihydroxyvitamin D_3_ treatment. LPS increased (*P* < 0.05) *CYP27B1, MT1A, MT2A, NOS2, TRX*, and *TXNRD1*. Interactions between LPS and 1,25(OH)_2_D_3_ (*P* < 0.05) were observed for *CYP24A1* and *TXNRD1* but not the other genes.

To further characterize the role of vitamin D signaling on antioxidant response, the effects of 25(OH)D_3_ on expression of known vitamin D pathway and antioxidant genes were assessed (Figure 2). As previously reported, LPS increased *CYP27B1* expression in monocyte cultures (Figure 2A). Likewise, IFN-γ induced *CYP27B1* in a dose-dependent manner (Figure 2A). *CYP27B1* encodes 1α-hydroxylase, which catalyzes conversion of 25(OH)D_3_ to 1,25(OH)_2_D_3_, indicating the potential for monocytes to increase 1,25(OH)_2_D_3_ synthesis when stimulated by IFN-γ or LPS. In contrast, 25(OH)D_3_ increased (*P* < 0.001) *CYP24A1*, which encodes the 24-hydroxylase that catalyzes inactivation of vitamin D (Figure 2B). Notably, IFN-γ and LPS decreased *CYP24A1* in the presence of 25(OH)D_3_ [IFN-γ dose × 25(OH)D_3_, *P* = 0.006; LPS dose × 25(OH)D_3_, *P* = 0.004; Figure 2B]. Further demonstrating vitamin D pathway activity, transcripts of *DEFB7* and *NOS2* were increased (*P* < 0.001) by 25(OH)D_3_ in the presence and absence of LPS or IFN-γ (Figure 2 C, D). However, IFN-γ decreased (*P* < 0.001) *DEFB7* in a dose-dependent manner (Figure 2C), even though it strongly increased *NOS2* expression (Figure 2D).

**Figure 2:**
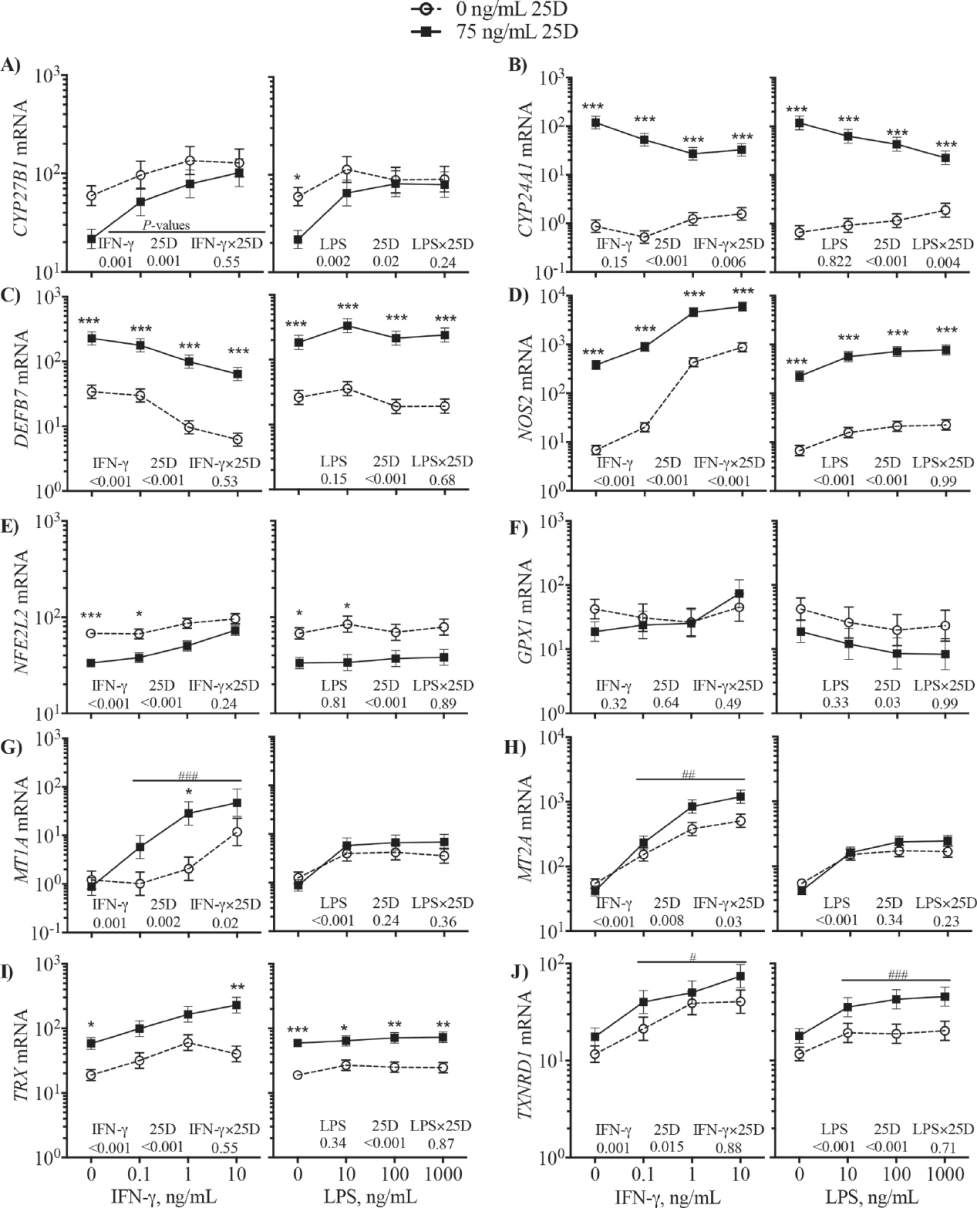
Monocytes from lactating Holstein cows (n = 4) were treated for 16 h with increasing concentrations of LPS or interferon-γ (IFN-γ) with 0 or 75 ng/mL 25-hydroxyvitamin D_3_ (25D) in a factorial arrangement. Transcripts for *CYP27B1* (A), *CYP24A1* (B), *DEFB7* (C), *NOS2* (D), *NFE2L2* (E), *GPX1* (F), *MT1A* (G), *MT2A* (H), *TRX* (I), and *TXNRD1* (J) were measured by quantitative PCR. Data represent the LSM ± SEM of ΔCt transformed by 2^−ΔCt^ and expressed as abundance relative to reference genes. The *P-*values for main effects of stimulant dose (IFN-γ, 0 to 10 ng/mL; LPS, 0 to 1,000 ng/mL), 25D (0 vs. 75 ng/mL), and their interaction (IFN-γ × 25D or LPS × 25D) are indicated on each plot. Tukey adjustment was made to account for multiple means comparison. *P < 0.05, **P < 0.01, ***P < 0.001: effect of 25(OH)D_3_ within dose of stimulant is significant. #*P* < 0.05, ##*P* < 0.01, ###*P* < 0.001: effect of 25(OH)D_3_ in stimulated cultures (0.1, 1, and 10 ng/mL IFN-γ or 10, 100, and 1,000 ng/mL LPS) is significant.

Although 1,25(OH)_2_D_3_ did not affect *NF2EL2* expression in our other experiment (Table 1), we found that 25(OH)D_3_ decreased (*P* < 0.001) *NFE2L2* expression (Figure 2E). In contrast, IFN-γ, but not LPS, increased *NFE2L2* in a dose-dependent manner, and the suppression of *NFE2L2* by 25(OH)D_3_ dissipated as IFN-γ increased (Figure 2E). Also, in contrast to our experiment with 1,25(OH)_2_D_3_, expression of *GPX1* was not affected by 25(OH)D_3_ in the presence of IFN-γ and was decreased (*P* = 0.02) by 25(OH)D_3_ in the presence of LPS (Figure 2F).

The metallothionein genes *MT1A* and *MT2A* were increased by 25(OH)D_3_ but the effect of 25(OH)D_3_ depended on stimulation (Figure 2G, H). Transcripts of each gene were strongly increased (*P* < 0.01) by IFN-γ or LPS stimulation. In the absence of stimulation, 25(OH)D did not affect *MT1A* or *MT2A*; however, 25(OH)D_3_ increased *MT1A* and *MT2A* in the presence of IFN-γ but not LPS stimulation [IFN-γ × 25(OH)D_3_ interaction, *P* < 0.05].

Transcripts of *TRX* were generally increased 3-fold (*P* < 0.001) by 25(OH)D_3_, and the effect of 25(OH)D_3_ did not depend on IFN-γ or LPS stimulation (Figure 2I). Stimulation with IFN-γ also increased *TRX* expression in a dose-dependent manner (*P* < 0.001), but LPS did not affect *TRX* expression. Likewise, 25(OH)D_3_ increased *TXNRD1* expression approximately 2-fold (Figure 2J). Both IFN-γ and LPS increased *TXNRD1* expression, and the effect of 25(OH)D_3_ appeared to become greater as the dose of LPS increased; however, the interactions between 25(OH)D_3_ and IFN-γ or LPS were not significant.

Our data collectively show a role for vitamin D in maintaining the redox balance of bovine monocytes. The oxidative environment generated by production of superoxides and nitric oxide in phagocytes is a key element in elimination of bacterial pathogens and redox signaling (Weiss and Schaible, 2015). Indeed, García-Barragán et al. (2018) reported that vitamin D-mediated NO production improved killing of *Mycobacterium bovis*. Generation of RNS, however, can lead to damage or impairment of infected tissues if not balanced by adequate supply of antioxidants (Trigona et al., 2006; Shi et al., 2018). As such, nutrients that have antioxidant properties or support antioxidant systems (i.e., vitamin E, Se, and Cu) are key nutrients in protection of cattle from bacterial diseases (Sordillo, 2016). Here, our data indicate that vitamin D also supports antioxidant activity in monocytes via increasing thioredoxin and metallothionein systems.

Physiological systems use several antioxidant mechanisms to combat the ROS produced from normal metabolism or oxidative bursts of phagocytes (Virág et al., 2019). Endogenous antioxidants such as the glutathione, metallothionein, and thioredoxin systems are known to protect host cells from ROS and maintain the redox balance (Itoh et al., 2005; Trigona et al., 2006). Previous work showed that 1,25(OH)_2_D_3_ increased thioredoxin reductase gene expression and enzyme activity in the human THP-1 monocyte cell line (Schütze et al., 1999). We hypothesized that vitamin D would increase antioxidant responses of bovine monocytes to counteract the pro-oxidant environment induced by TLR and IFN-γ signaling. Collectively, our data show that vitamin D signaling contributes to induction of endogenous antioxidant systems of bovine monocytes. For example, in the presence of IFN-γ stimulation, 25(OH)D_3_ increased antioxidant potential and expression of metallothionein genes. Furthermore, 25(OH)D_3_ increased expression of *TRX* and *TXNRD1* regardless of stimulation. Thioredoxin and metallothionein have protective roles against NO-induced cell damage (Schwarz et al., 1995; Ferret et al., 2000). Thus, we speculate that increased antioxidant activity from thioredoxin and metallothionein may serve as a protective factor against vitamin D-induced NO production in monocytes.

Certainly, our data do not rule out other antioxidant systems because we did not measure specific antioxidant activities. Glutathione peroxidase activity protects against nitric oxide-induced damage of mammary epithelial cells (Shi et al., 2018) and, in other species, vitamin D signaling increases glutathione concentrations (Jain and Micinski, 2013; Xu et al., 2015). Here, treatment of monocytes with 1,25(OH)_2_D_3_ increased *GPX1* expression, but the opposite was observed with 25(OH)D_3_ treatment. We speculate the conflicting results stem from the timing of each metabolite's actions. For instance, 1,25(OH)_2_D_3_ can act immediately in the cell, whereas 25(OH)D_3_ must be converted to 1,25(OH)D_3_ by CYP27B1. Keeping in mind that we measured steady-state transcript abundance at only one time point, it is quite likely the transcript abundance for each gene was not reflective of respective antioxidant activities. Furthermore, posttranslational mechanisms exert substantial control over antioxidant activities of glutathione and thioredoxin systems (Asahi et al., 1995; Rundlöf and Arnér, 2004). Thus, further experiments are needed to assess the effects of vitamin D on specific antioxidant activities in bovine immunity.

The ability of 25(OH)D_3_ to support the antioxidant potential of monocytes seemed to depend on stimulation, particularly that of IFN-γ, indicating that the vitamin D and IFN-γ pathways work together to maintain the redox balance of monocytes. For instance, dROM was less in cultures treated with IFN-γ and 25(OH)D_3_ compared with those treated with 25(OH)D_3_ alone, and upregulation of metallothionein genes depended on IFN-γ stimulation. We also observed that, apart from 25(OH)D_3_, IFN-γ was a potent stimulator of metallothionein and thioredoxin genes.

The dependency of 25(OH)D_3_ effects on antioxidant responses may be explained in part by the transcription factor NFE2L2, a key factor in the activation of cellular antioxidant defenses, including induction of *MT1A, MT2A, TRX*, and *TXNRD1* (Sakurai et al., 2005; Fujie et al., 2016). In our experiments, 25(OH)D_3_ downregulated expression of *NFE2L2* but the effect of 25(OH)D_3_ on *NFE2L2* became less pronounced with increasing IFN-γ. Accordingly, we observed the same pattern of responses for antioxidant potential and metallothionein genes. The interaction between IFN-γ and vitamin D also may occur at the point of NFE2L2 protein activity and stability, which we did not measure. To our knowledge, direct binding of the VDR to the metallothionein and thioredoxin promoters in cattle or other species has not been reported. On the other hand, Dai et al. (2019) reported that 1,25(OH)_2_D_3_ increased antioxidant responses by increasing NFE2L2 translocation to the nucleus. Likewise, Tao et al. (2019) reported that 1,25(OH)_2_D_3_ decreased NFE2L2 ubiquitination. Those interactions between the VDR and NFE2L2 provide a plausible explanation for how vitamin D may increase metallothionein and thioredoxin responses despite downregulation of *NFE2L2* expression by 25(OH)D_3_ treatment. Future work should explore the interactions between NFE2L2 and VDR proteins in regulation of antioxidant responses in cattle.

The implications of our findings are significant in understanding the benefits of vitamin D in transition cow health and production. The health benefits that were observed from supplementing prepartum cows 25-hydroxyvitamin D_3_ (Martinez et al., 2018) may involve increased antioxidant potential of immune cells, in addition to the previously reported antimicrobial actions of vitamin D in bovine immunity (Yue et al., 2017; García-Barragán et al., 2018). In theory, the capacity of vitamin D signaling to increase endogenous antioxidant mechanisms of immune cells will limit the degree of oxidative stress and subsequent tissue damage that occurs from inflammatory insults, such as those of the uterus and mammary glands of postpartum cows that are susceptible to bacterial infections. Consequently, biomarkers for oxidative stress may be key outcomes to assess for the effects of vitamin D treatments in cattle.
